# The Role of Moderate Aerobic Exercise as Determined by Cardiopulmonary Exercise Testing in ALS

**DOI:** 10.1155/2018/8218697

**Published:** 2018-01-31

**Authors:** Anna Caroline Marques Braga, Anabela Pinto, Susana Pinto, Mamede de Carvalho

**Affiliations:** ^1^Translational and Clinical Physiology Unit, Molecular Medicine Institute, Faculty of Medicine, University of Lisbon, Lisbon, Portugal; ^2^Department of Physical Medicine and Rehabilitation, Hospital Santa Maria-CHLN, Faculty of Medicine, University of Lisbon, Lisbon, Portugal; ^3^Department of Neurosciences, Hospital Santa Maria-CHLN, Lisbon, Portugal

## Abstract

**Introduction:**

The efficacy of cardiopulmonary exercise testing (CPET) to determining exercise intensity has not been established in Amyotrophic Lateral Sclerosis (ALS). We studied this intervention.

**Methods:**

We included 48 ALS patients randomized in 2 groups: G1 (*n* = 24), exercise intensity leveled by CPET; G2 (*n* = 24), standard care limited by fatigue, during 6 months. ALS functional scale (ALSFRS-R) and forced vital capacity (FVC) were performed every 3 months; CPET was done at admission (*T*1) and 6 months later (*T*2). We registered oxygen uptake, carbon dioxide output, and ventilation at anaerobic threshold and at peak effort. Primary outcome was functional change. We used parametric statistics for comparisons and multiple regression analyses to identify independent predictors of functional decline.

**Results:**

At *T*1 both groups were identical, except for higher FVC in G1 (*p* = 0.02). At *T*2, ALSFRS-R was higher (*p* = 0.035) in G1. Gas exchange variables at *T*2 did not change in G1 but had significant differences in G2 (*p* < 0.05). Multiregression analyses showed the Spinal ALSFRS-R slope and Intervention group (*p* < 0.001) as significant predictors of ALSFRS-R at *T*2.

**Conclusion:**

Aerobic exercise defined by CPET is feasible and can improve functional outcome in ALS. This trial is registered with Clinical trials.gov ID: NCT03326622.

## 1. Introduction

Exercise is widely recommended to the general population due to its benefits to health and wellbeing. It improves the cardiovascular, respiratory, musculoskeletal, and endocrine functions and leads to psychological wellbeing. The role of exercise in the elderly, often with functional limitations and high risk of falls, is not yet completely clarified [1]. In Amyotrophic Lateral Sclerosis (ALS), robust evidence about its risks and benefits is not established and its putative neuroprotective role is still controversial [2, 3]. Disease-specific guidelines as general exercise recommendations, which are part of standard of care for ALS, with instructions for stretching, range of motion exercises, balance, and physical activity, are based on preclinical data, small human studies, and research on exercise in other neuromuscular diseases. Recent and increasing evidence in animal models and human studies reinforces the benefits of an exercise program suggesting that moderated endurance exercise can delay disease onset and increase survival [1–6]. Aerobic exercise comprises a myriad of forms, and it is generally performed at a moderate level of intensity with longer duration than its counterpart: the anaerobic or strengthening exercise. The former refers to the use of oxygen to adequately meet energy demands during exercise via aerobic metabolism, which is critically related to the cardiorespiratory and vascular system's capacity to supply oxygen to the muscles, and the ability to clear carbon dioxide from the blood via the lungs [4]. When the intensity of the exercise exceeds the rate of oxygen supply to the muscles by the cardiovascular and respiratory systems, lactate builds up and quickly makes it impossible to continue the exercise. The starting point of the exponential increase of lactate during a cardiopulmonary exercise testing (CPET) is the anaerobic threshold (AT). In ALS, AT may occur sooner than expected due to respiratory muscle weakness. However, no useful clinical symptom or sign is known as a marker of the AT and it can only be determined by direct measures of gas exchanges analysis through a cardiopulmonary exercise testing (CPET). On the other hand, the gap between AT and the respiratory compensation point (RCP), point of exercise intensity above which only anaerobiosis occurs, the training zone, may become narrowed or difficult to determine. In these circumstances, a safe limit is usually accepted by adding 10 to 20% of the work intensity at AT that has to be uncovered. To overcome the difficulties and help the clinician to define the limits of the training zone and thus prescribe a moderate exercise program, the measurement of the aerobic capacity (VO_2_) at anaerobic threshold (VO2_AT_), at the RCP or at peak of effort (VO_2pk_), can be done through the use of CPET with gas exchange analysis. The training zone can also be set from the lowest nadir of the curve VE/VCO2 [5].

In addition, overtraining precautions are needed to avoid cramps, fasciculation, myalgia, prolonged postexercise fatigue, or soreness that are usually related to excessive neuronal hyperactivity and are clinical useful indicators of overwork. Postexercise fatigue should not interfere with daily life activities. If a patient has fatigue or pain that lasts longer than 30 minutes after exercise, the program needs to be reduced and modified [6, 7]. Moreover, as the etiology of nerve cell death in ALS is complex and multifactorial, with excitotoxic mechanisms playing a role together with reduced oxidative metabolism [8], it is relevant to evaluate the effects of a moderate aerobic exercise with controlled intensity determined by CPET and its role on the functional status in ALS patients versus standard care. This work assessed these effects between baseline and six months of follow-up (primary outcome) and additionally explored the performance of CPET variables throughout the study (secondary outcome).

## 2. Materials and Methods

### 2.1. Study Design

We carried out a prospective, single-blinded, quasi-randomized controlled trial, including 48 consecutive ALS patients referred to the Rehabilitation Department of our Hospital by neurologists who were blinded to the study. In a quasi-randomized study participants are allocated to either the intervention or control groups by using a random allocation sequence by alternation between groups [9]. In our study, patients were allocated to two groups, based on geographical residence: Group 1 (G1, *n* = 24) included ALS patients with residence within the hospital outskirts; Group 2 (G2, *n* = 24) included patients with residence outside hospital area limits. All patients in both groups were ambulatory and able to perform CPET before the admission (*T*1), but only 6 patients in G2 performed it mainly due to agenda and transport constraints.  Table 1 describes the inclusion and exclusion criteria of the trial.

### 2.2. Exercise Training Protocol

Patients in G1 and G2 performed a standard care exercise program, as determined by the American Academy of Neurology guidelines [10]. It included daily exercises, such as Range of Motion (ROM) exercises, limbs relaxation, trunk balance, and gait training. While patients in G2 performed the program at home or at other rehabilitation units, G1 patients were supervised in our Unit and, in addition to the standard care, they also performed an aerobic exercise protocol two times per week on a treadmill, with training zone determined by CPET. The patient effort was considered as moderate intensity. When the training zone was not identified due to undetermined RCP, it was leveled-up 20% of the work rate at AT achieved in the CPET. Noninvasive ventilation (NIV) was added as needed for both groups and adjustments to the aerobic exercise program were made in accordance with cardiorespiratory responses of each patient in G1 [11–14]. Body weight supporting system (BWSS) was used for patients with minimal lower limb weakness in G1. No BWSS was used during the training sessions in the G2.

### 2.3. Assessments

All patients were assessed at first visit (diagnostic visit,* T*0), at study entry (*T*1), and 6 months after (*T*2) as follows.

#### 2.3.1. Revised ALS Functional Rating Scale (ALSFRS-R)

All patients were evaluated with the revised ALS Functional Rating Scale (ALSFRS-R) [15]. This tool rates the functionality of the ALS patients in performing activities involving 4 different areas and subscores, bulbar, upper limb, lower limb, and also the respiratory function, each of its questions rated from 0 (total inability) to 4 points (normal function). The last three questions address the respiratory function (dyspnoea, orthopnea, and respiratory insufficiency) [15].

#### 2.3.2. Respiratory Function Tests (RFT) and Nocturnal Pulse Oximetry (NPO)

Forced Vital Capacity (FVC) and NPO were performed as described elsewhere [16, 17]. The percentage of the predicted value of FVC was recorded for posterior analyses. RFT including maximal inspiratory and expiratory pressures, phrenic nerve conduction studies and oxygen saturation provided by NPO in terms of mean percentage of oxygen saturation (% SpO2), the percentage of recording time with oxygen saturation lower than 90% (Sat < 90%), and the number of oxygen desaturations per hour (ODI) were used to assess the need and appropriate time for nocturnal NIV adaptation in both groups [17].

#### 2.3.3. Cardiopulmonary Exercise Testing (CPET)

CPET was performed at study entry and 6 months later (*T*1 and* T*2), using a treadmill (Woodway®) coupled with a gas exchange analyzer (METALYZER® 3B) with ergo-spirometry system using a breath-by-breath technology developed by CORTEX® systems. Data were extracted and analyzed with application software Metasoft® Studio. The testing was customized and tailored to achieve symptom-limited exercise. A ramp modified protocol with increments of 5–15 Watts/minute, with a duration of 8 to 12 minutes, including 3-4 minutes for warm-up and cooling down. Patients were continuously monitoring with pulsed oximetry and three ECG leads [18].

The peak effort was considered as achieved. We interrupt the test when the participants presented some of the following situations: reaching 75% of the predicted maximum heart rate (220-age), reaching 55–65% of the predicted VO_2_ maximum for age, gender, height, and weight, and/or reaching fatigue evaluated by the Borg modified perceived scale or presented loss of neuromuscular performance. Other end-testing flags were complaints of lower limbs' pain, dyspnoea, presence of desaturation (SpO_2_ ≤ 88%), or the achievement of RCP [19]. All patients achieved the anaerobic threshold.

The CPET variables analyzed were oxygen uptake expressed in L/min (VO_2_), in percentage of predicted, or in metabolic equivalents (METs) at peak effort, anaerobic threshold (AT), and the respiratory compensation point (RCP) when achieved, Dioxide Carbon output in L/min (VCO_2_) and minute ventilation in L/min (VE).

### 2.4. Data Analysis and Statistics

Frequency distributions (median and interquartile) were calculated for age at study, disease duration, and categorical variables. Time measurements are expressed in months. The other continuous variables are presented with means ± standard deviation (m ± SD) and were expressed in absolutes values: Age at onset; disease duration* T*0-*T*1, % FVC predicted, CPET variables (VO_2_ peak, VO_2AT_, MET's, and VE), ALSFRS-R score, its subscores (bulbar, spinal, and respiratory), and respective slopes. ALSFRS-R slopes between* T*0-*T*1 and* T*1-*T*2 were calculated by subtracting the ALSFRS-R score difference between (*T*0-*T*1) and (*T*1-*T*2) divided by time between evaluations.

To assess the normality and variance, Kolmogorov-Smirnov test was performed. Parametric tests were used to explore differences between groups and subgroups regarding total ALSFRS-R, its subscores and slopes, % FVC, and CPET variables. Categorical variables (gender, region of onset, group, and use of NIV) were transformed from dummy to metric variables to be submitted to stepwise multivariate linear regression analyses. We inputted the means for missing data points for both groups. Multiple regression model was applied to identify independent predictors of functional change at* T*2. All tests were 2-tailed, with significance set at 0.05 and power 0.7 (G^*∗*^. Power version 3.1.9.2). SPSS package software v. 22 was used.

### 2.5. Ethical Committee

The present study was submitted and approved by the Institutional Ethical Committee based on the national legislation (Reg. Number 287/13 – 14 June 2013). All patients signed an informed consent.

## 3. Results

**Figure 1:**
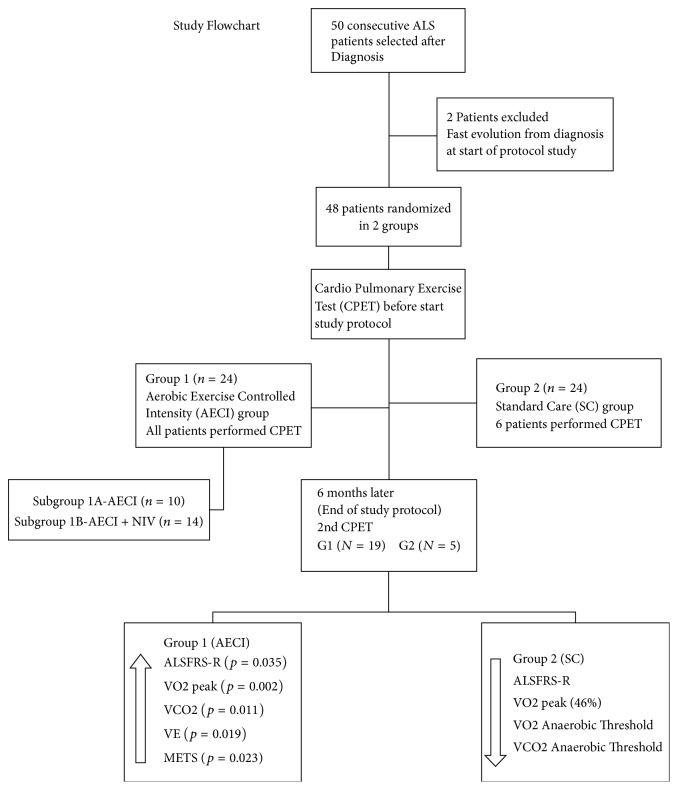
At the end of study we can identify the main findings between groups. The arrows indicate the direction of the significant differences in G1 when compared with standard care group G2. The VO2 peak in G2 reduced 46% since* T*1. AECI: aerobic exercise with controlled intensity and NIV: non-invasive ventilation.

We included 48 patients, 32 men. Disease duration from onset was similar between G1 (median = 9.50 months; IQR [25%–75%] 5.25–11.75) and G2 (median = 9.00 months; IQR [25%–75%] 5.25–12.00) (*p* = 0.7). The median age at study was 60.5 years for G1 (IQR [25%–75%] 54.25–76.25) and 63.0 years for G2 (IQR [25%–75%] 59.25–68.50) with nonsignificant difference (*p* = 0.4). Age at onset, gender, region of onset, and ALSFRS-R and its subscores were equivalent between groups. % FVC predicted was significantly lower in G2 at entry. The study flow chart is shown in Figure 1. Clinical and demographic characteristics are summarized in Table 2. The sample presented a normal distribution. Twenty-four patients were randomized to G1, the active exercise group monitored in-house and 24 to G2, the control group.

At* T*0 (diagnosis), G2 had a higher percentage of older women with bulbar onset – 30% versus 12% in G1 though a nonsignificant difference; ALSFRS-R total score and its subscores were also nonsignificant. At the start of study (*T*1) there was no difference between subscores (Bulbar score: *p* = 0.14; Spinal score: *p* = 0.12; Respiratory score: *p* = 0.93). All patients were stable with oxygen saturation (SpO_2_) ≥ 95%.

**Figure 2:**
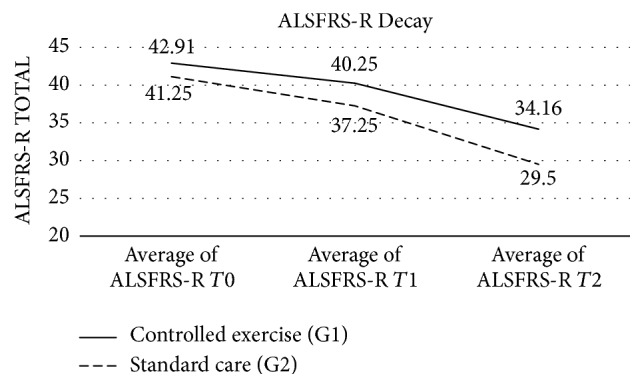
Slope of ALSFRS-R total score between* T*0,* T*1, and* T*2 for both groups.

At end of study (*T*2), ALSFRS-R was significantly higher in G1 (*p* = 0.035). There was a nonsignificant trend for a reduced subscores slopes in G1. To determine whether there was a difference on the decline of ALSFRS-R between groups, we calculated the slope of ALSFRS-R total between* T*0 and* T*1 (*p* = 0.19; CI 95% [−0.69–0.14]) and between* T*1 and* T*2 (*p* = 0.36; CI 95% [−0.86–0.32]), and the effect size (*d*) = −0.26 showed a small but positive effect favoring the exercise group G1 (see Figure 2).

### 3.1. Predictors of ALSFRS-R Total at End of Study: Multiple Linear Regressions Analyses

We investigated the relationship between the functional score achieved at end of study and the following independent variables: age at study, gender, region of onset, use of NIV, group of intervention, and slopes of ALSFRS-R total. The stepwise multiple linear regression analysis adjusted to FVC at* T*1 showed that Bulbar Slope (*B* = −5.084; *p* = 0.12), Spinal Slope (*B* = −6.152; *p* < 0.001), and Group of intervention (*B* = 3.833; *p* = 0.021) were independent predictors. Together they explained 54.3% of the variance of the achieved ALSFRS-R score at end of study with adjusted *R*^2^ = 0.51. The regression model was significant (*p* < 0.001), and analyses with Durbin Watson test showed that the data had no autocorrelation. We found an effect size *f*^2^ = 1.04 favoring the intervention group.

### 3.2. Influence of Use of Noninvasive Ventilation on the ALSFRS-R at* T*2

**Figure 3:**
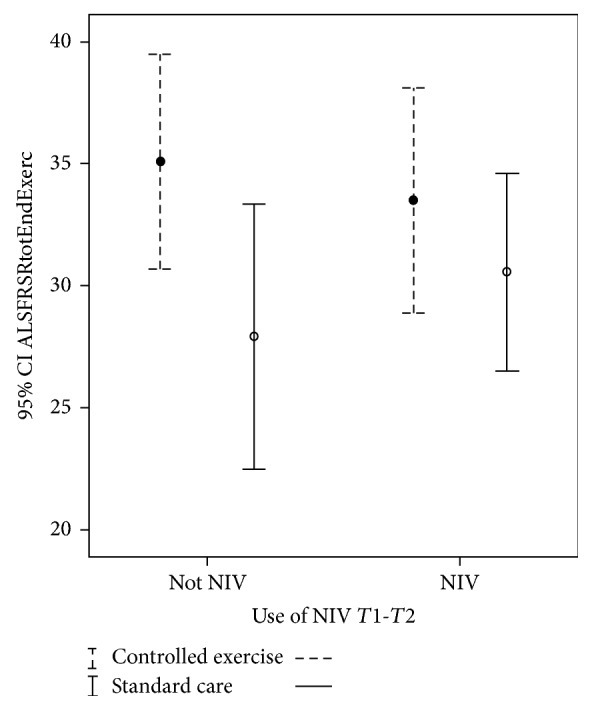
Influence of use of NIV on ALSFRS-R at end of study, Confidence Interval 95% (−3.08–6.04).

Subgroup 1A (*n* = 10) did exercise without NIV and Subgroup 1B (*n* = 14) used NIV during exercise sessions. G2 used NIV as needed. About 50% of patients in both groups used NIV (Table 2). However, a simple linear regression analysis showed no influence on ALSFRS-R change at* T*2 by the use of NIV (*p* = 0.7, *R*^2^ = 0.02) (Figure 3).

### 3.3. Performance of Cardiopulmonary Exercise Testing (CPET) Variables during the Study

In G1 all patients completed the exercise program, but only 19 (79%) were gait-independent at the second CPET evaluation. In G2, 6 patients performed a first CPET and only one patient of those did not perform the second CPET. Out of the remaining patients (18), only six of them had had gait-independence at* T*2 (29%).

### 3.4. CPET Variables at Peak Effort

**Figure 4:**
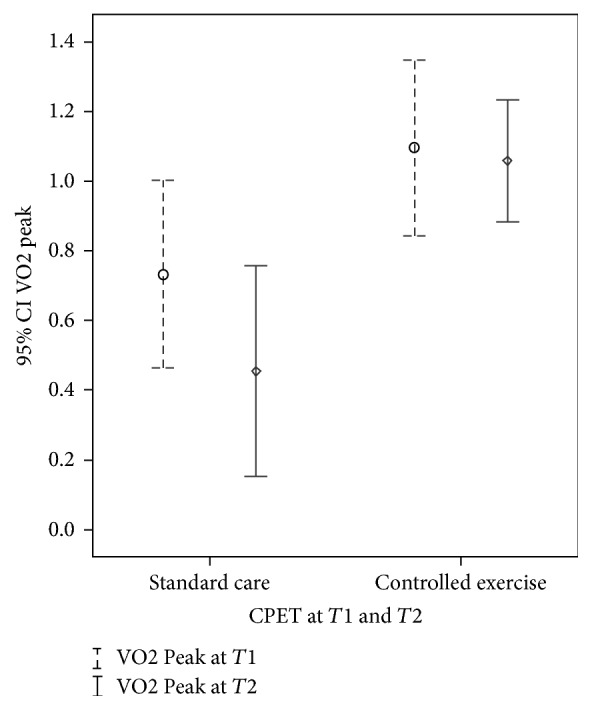
VO2 Peak at* T*1 (*p* = 0.13, [CI: −0,78–0,11]) and at* T*2 (*p* = 0.002, [CI: −0,96–−0,25]).

We found no differences between groups regarding CPET variables (VO_2_, VCO_2_, VE, METs, and RCP) both at AT and peak effort at* T*1. The average peak VO_2_ in % of predicted for G1 was 60.8% (±21.2) and G2 was 44.16% (±12.45) (*p* = 0.07). As all the patients presented to CPET in* T*1 and* T*2 indicated equal variances on the homogeneity test, that allowed us to assume the implications to the differences between groups with different sample sizes. At* T*2 there were significant differences between groups related to VO_2peak_ (*p* = 0.002), METs (*p* = 0.023), VCO_2_ (*p* = 0.011), and VE (*p* = 0.019) (see Table 3). The confidence intervals with significant differences at end of study for VO_2peak_ are presented in Figure 4.

### 3.5. CPET Variables at Anaerobic Threshold

**Figure 5:**
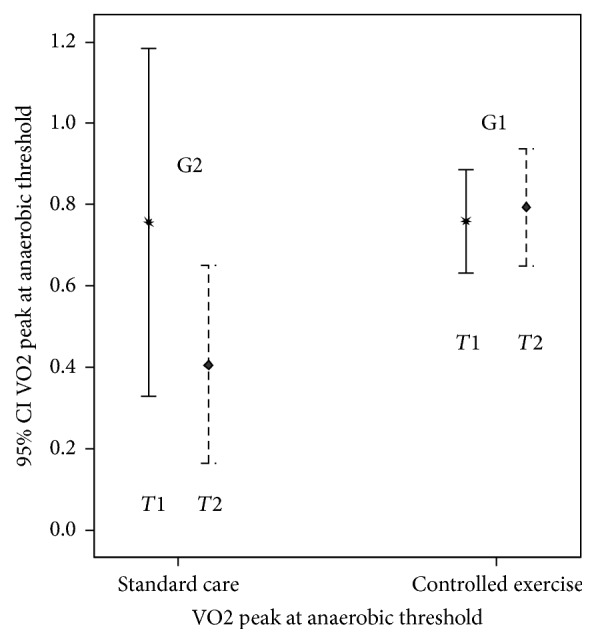
VO2 at the Anaerobic Threshold,* T*1 (*p* = 0.8, [CI 95%: −0,23–0,29]) and* T*2 (*p* = 0.02, [CI 95%: −0,70–−0,06]).

Regarding the work capacity on the anaerobic threshold, there were no significant differences at entry, but significant differences (*p* < 0.05) at* T*2 for VO_2_ and VCO_2_. These variables were significantly higher in G1 than in G2 (Table 4). The confidence interval with significant differences at end of study for VO_2AT_ is presented in Figure 5.

### 3.6. Aerobic Capacity and ALSFRS-R at End of Study

While patients in the G1 presented a stable condition regarding aerobic capacity, anaerobic threshold, and ventilatory capacity, the patients in the G2 showed significant decrease for the same aspects between *T*1 and *T*2 (Tables 2 and 3). Peak VO_2_ decreased 10.25% in G1 and 46% in G2. There were significant differences on the oxygen uptake, CO_2_ output, and ventilatory capacity, with a very high effect size (*d* = 1.99) analyzed by Cohen's* d* on peak VO_2_ between groups. In addition, we found a significant and positive correlation between ALSFRS-R total score at end of study and peak VO_2_, METS, VCO_2_, and VE (Table 5), but no correlation of ALSFRS-R at* T*1 with the same variables.

## 4. Discussion

Nowadays, there is no strong evidence showing a potential harmful effect of exercise in ALS. The unpredictable progression of the disease, the different phenotypes, the frequent methodological shortcomings, and ethical issues affect most of the studies.

A weak muscle can be damaged if overworked, which can easily happen in ALS as it is already functioning close to its maximal limits [20]. This is the reason why some experts have discouraged exercise programs in ALS. These all make daily activities harder to do [21].

Moderate exercise may have a beneficial effect on free-radical balance and improve muscle fiber oxidative metabolism, with potential impact on excitotoxicity [22]. The protection against oxidative stress has special significance as in ALS the motor neurons are particularly susceptible to oxidative damage [23].

On top of this, if defective mitochondrial energy metabolism plays a role in cell death in neurodegenerative disorders and exercise may trigger added neuron excitability, we considered it of utmost relevancy to evaluate the effect of a moderate exercise program with work intensity close to the AT precisely determined by CPET.

To the best of our knowledge, only three studies have been published on aerobic exercise capacity in ALS, with exercise intensity established by determination of CPET [24–26]. All of them showed a reduced peripheral O_2_ utilization suggested to be consistent with physical deconditioning as the main cause of impaired exercise capacity in ALS, possibly related to impaired oxidative metabolism, early AT, and low peak oxygen uptake. The latter was not found in the other neuromuscular disorders. However, none of those studies evaluated the effect of a moderate exercise program on oxygen uptake around AT throughout disease progression. Our study is relevant due to the probable implications regarding the potential benefit of the rigorous exercise intensity prescription determined in the CPET and the risk of unsupervised exercise above the anaerobic threshold [27]. Indeed, there are no clinical determinants of AT such as the time limit to fatigue, work intensity to fatigue, or ventilatory responses; in addition, peak oxygen uptake (peak VO_2_) cannot be used to estimate anaerobic capacity due to the large contribution of intraindividual variability [28].

This is the first exercise trial applying a moderate exercise protocol with intensity rates precisely defined through gas exchanges measures. Despite the limitations of a small sample, even over the apparent heterogeneity at the beginning of the protocol, but not at diagnosis, we counter these differences by recognizing that patients in G2 had a larger percentage of older women with bulbar onset who were expected to have a poorer prognosis regarding bulbar slopes and scores in G2. And patients in G1 had a larger proportion of spinal onset, who were expected to present a more progressive rate of decline of ALSFRS-R spinal scores or slopes. However, neither of these assumptions was observed, most likely due to the effect of exercise program that the two groups were instructed to follow during this period of time, showing no differences in the spinal, bulbar, or respiratory slopes at* T*2 (Table 1). Using a multiple linear regression model we found the group of intervention as a significant independent predictor (*B* = 3.833; *p* = 0.021).

These observations taken together with the significant difference in ALSFRS-R spinal subscore favoring G1 patients at* T*2 (*f*^2^) = 1.04 and the mean difference of functional decline expressed on the ALSFRS-Total score between groups after 6 months also showing a small but positive effect favoring the exercise group (*d*) = 0.26 (Figure 2) strengthen our refutation of the heterogeneity of the sample population.

Our results concur with the recent study by Lunetta et al. [29] that also showed that a strictly monitored moderate exercise program may significantly reduce motor deterioration in ALS patients. Interestingly they were not able to improve survival, an essential point to demonstrate a neuroprotection effect, and the authors were not clear regarding the definition of moderate exercise

Actually, the possibility of a muscle fiber to increase its size and becoming stronger while maintaining endurance capacity depends primarily on a set of different factors, such as the application of appropriate stimuli (i.e., sustained contractile activity combined with short, powerful mechanical loading), availability of the essential substrates, the capacity to increase oxygen transport (e.g., by improving heart and lung function or angiogenesis, hematocrit and myoglobin), and prevention of tissue hypoxia with chronically reduced cellular energy status.

Moreover, the cellular oxygen supply can be improved by increasing the capillarization, the hematocrit, or myoglobin concentration [30], in which the regulation involves the hypoxia-inducible factor-1 (HIF-1*α*). The HIF-1*α* mediates the expression of erythropoietin and angiogenic growth factors, such as vascular endothelial growth factor (VEGF), known to be implicated in ALS pathogenesis [31]. VEGF can be increased in serum concentrations in ALS patients both by moderate exercise and noninvasive ventilation as previously shown by our team [32]. Thus, we took it into consideration and applied a moderate exercise program and NIV as needed in order to enhance an hypothetical neuroprotective effect, such as suggested by Dal Bello-Haas and Florence, 2013 [33].

Unexpectedly, NIV did not exert any influence on ALSFRS-R at* T*2 (*p* = 0.46, *R*^2^ = 0.02) (Figure 3). Given the well-known effects of NIV on survival, quality of life, exercise tolerance, and sleep quality, the most likely explanation is related not only to the very similar approach of initiation of NIV in both groups but also to the short timeframe of observation.

No doubt these factors will also have to be considered in further studies, when addressing the major issue of neuroprotection and survival benefit. However, whether these results corresponded just to an expected initial distal plasticity as shown by Blizzard and colleagues, 2015 [34], or a positive effect on neuroprotection, as suggested in a previous study of our team [35], still remains to be explained and will be the focus of a future longitudinal study that due to the extensive and expensive nature of the needed evaluations will justify a multicenter trial.

Regarding the performance of CPET variables during the study, the anaerobic threshold (AT), also called the ventilatory threshold (VT), is an index used to estimate exercise capacity. It constitutes a reliable and reproducible index of submaximal exercise intensity that is defined as the highest VO_2_ that can be sustained without developing a lactic acidosis, a response that is generally observed at 40 to 60% of peak VO_2_ independently of patient-motivation.

A key utility of AT is that it provides information at a submaximal level of exercise intensity (i.e., it does not require a physiologically maximal exercise effort) and is also considered more consistent with a patient's ability to perform daily activities, especially because exercising beyond the AT for sustained periods eventually results in fatigue.

In addition, we used the most common method that entails the graphing values of VCO_2_ versus VO_2_ to identify the AT as the point where there is a shift in slope along a line of identity between these gas measurements (modified V-slope method) [5]. Mean values of oxygen uptake at AT expressed in % of achieved peak VO_2_ at* T*1 was 69%, which allowed us to cast some doubts over the deconditioning clinical situation of our patients in both groups at entry in the study. At* T*2, patients in G2 showed significant differences with a very rapid decrease of VO_2(AT)_, though it happened in an even higher percentage (88%) of VO_2_ peak probably due to a primarily neurogenic impairment. On the other hand, these results also show that deconditioning was not the main reason of poor performance, usually identified with low VO_2_ and early AT, though it still is a common point of view.

Together with a respiratory compensation point (RCP) > 0.80 (see Table 3) in both evaluations and groups, it shows not only the existence of peripheral muscle underutilization of oxygen as described by other authors, but specifically a primarily impairment of muscle performance probably due to atrophy and loss of muscle bulk with a late increase in lactate and low VO_2_, exactly the opposite results for a mitochondrial myopathy with an early increase in lactate, combined with a very low VO_2_ peak, as shown by Takken and colleagues, 2010 [36]. Likewise we recognized a primarily neurogenic impairment instead of deconditioning.

Our study does not address the important issue of muscle oxygen extraction impairment, a dysfunction recently described in ALS [31]. In future studies this evaluation should be added to investigate the impact of exercise in ALS.

Peak VO_2_ is an important metric because it defines the limits of the cardiopulmonary system. Although commonly expressed in L/min, this value naturally increases as body mass increases. To better facilitate intersubject comparisons, peak VO_2_ is usually normalized and expressed in ml/Kg/min. However, the relationship of peak VO_2_ and weight is not linear with inherent imprecision associated with weight-normalized values; thus we recorded VO_2_ either in L/min or in percentage of predicted values or in METs.

Remarkably, our results showed a significantly more stable course of peak VO_2_ in patients of G1 suggesting that exercise prescribed and performed according to the CPET evaluation has a positive impact on functional decline. However, we cannot discard the effect of a supervised exercise program with expert physiotherapists also able to modify the work intensity according to individual physiologic responses at each session.

Moreover, it is not possible to exclude a bias effect due to a better respiratory function, FVC in G1, though its measurement is sometimes problematic in patients with bulbar weakness [32]. Indeed, the lower FVC in G2 patients was likely due to an insufficient tight seal with pursed lips for accurate measurement. Nevertheless, we adjusted our results to the FVC by a stepwise multiple linear regression analysis and found an effect size *f*^2^ = 1.04 favoring the intervention group strengthening our principal conclusion.

These findings support our hypothesis that aerobic exercise with control of intensity leveled by CPET can be safe and beneficial for ALS patients prolonging ambulatory skills.

Indeed, exercise, when prescribed and supervised appropriately, may be physically and psychologically important for people with ALS, especially in the earlier stages of the disease and before significant muscular atrophy occurs. Although it may not improve the strength of muscles already weakened by ALS, strengthening exercises with low to moderate weights and aerobic exercises such as swimming, walking, and bicycling, at submaximal levels may be important components of an overall management plan [33]. An exercise prescription in a rehabilitation program for ALS patients should follow an assessment by CPET with aerobic capacity measurements and be performed under strict and competent supervision.

## 5. Conclusions

Moderate exercise protocol with CPET evaluations can be safe and beneficial and should be considered in the multidisciplinary approach to ALS patients.

## Figures and Tables

**Table 1:** Inclusion and exclusion criteria of the present study.

Inclusion criteria

Age between 18 and 90 years
Diagnosis of definite, probable, or probable laboratory supported ALS
Disease duration from first symptoms between 6 and 24 months
ALSFRS-R ≥ 30
FVC (% predicted) ≥ 70%

Exclusion criteria

Other medical conditions, like cardiac insufficiency and lung disorders or others conditions limiting exercise training
Heavy smoking habits with laboratorial evidence of significant bronchial constriction
Signs of associated dementia or psychiatric disorders

*Note*. None of the patients were on tube feeding, invasive or noninvasive mechanical ventilation at admission of study protocol (*T*1).

**Table 2:** Clinical and demographic characteristics of the ALS patients at diagnosis (*T*0), admission to the study protocol (*T*1), and the end of study (*T*2). *T*-Test: mean and standard deviation values for both groups.

	Group 1 (*n* = 24)	Group 2 (*n* = 24)	*p* value
Male (%)	18 (75%)	14 (59%)	0.20
Spinal onset form (%)	21 (88%)	17 (70%)	0.12
Use of NIV *T*1-*T*2 (Yes – No)	14Y/10N	13Y/11N	0.75
Age at onset (years)	63.21 (±13.0)	62 (±12.06)	0.42
Disease duration (*T*0-*T*1) (months)	10.8 (±6.5)	10.79 (±7.7)	0.80
% FVC predicted (*T*1)	99.64 (±21.8)	80.0 (±21.0)	**0.002**
ALSFRS-R total score (*T*0)	42.92 (±3.51)	41.13 (±4.83)	**0.14**
ALSFRS-R total score (*T*1)	40.25 (±5.00)	37.25 (±4.9)	0.042^*∗*^
ALSFRS-R total score (*T*2)	34.1 (±7.1)	29.5 (±7.7)	**0.035**
ALSFRS-R Tot sc. slope (*T*1-*T*2)	1.01 (±0.92)	1.28 (±1.10)	0.36
ALSFRS-R bulbar slope (*T*1-*T*2)	0.15 (±0.24)	0.18 (±0.25)	0.62
ALSFRS-R spinal slope (*T*1-*T*2)	0.66 (±0.64)	0.75 (±0.97)	0.68
ALSFRS-R respiratory slope (*T*1-*T*2)	0.20 (±0.26)	0.30 (±0.32)	0.23

G1: controlled exercise Group, G2: standard care group, *T*0: at diagnosis; *T*1: at start of to study; *T*2: end of study; Sp: spinal onset, Bb: bulbar onset; use of NIV*T*1-*T*2: use of noninvasive ventilation during period of study; % FVC predicted *T*1: % forced vital capacity predicted at start of study (*T*1);  ALSFRS-T_Total Diagnosis_: ALSFRS-R total at diagnosis moment; ALSFRS-R_total  T2_: ALSFRS-R total at end of study; slope ALSFRS-R_Total  T1-T2_: slope ALSFRS-R total between start and end of exercise protocol (*T*1-*T*2); significant results (*p* ≤ 0.05) are represented in bold. ^*∗*^*ALSFRS-R total score on T1* (*Subscores between G1 and G2 (NS): bulbar score: p* = 0.14*; spinal score: p* = 0.12*; Respiratory score: p* = 0.93).

**Table 3:** Cardiopulmonary exercise testing measurements on the peak of effort. *T*-test.

CPET variables (L/min)	Group 1 exercise (*n* = 24) Mean (±S.D)	Group 2 standard care (*n* = 6) Mean (±S.D)	*p* value (between groups G1-G2)
VO_2Predicted_	2.06 (±0.58)	1.87 (±0.54)	0.47
% VO_2Predicted_	60.8 (±21.2)	44.16 (±12.45)	0.07
VO_2peak_ *T*1	1.17 (±0.50)	0.83 (±0.32)	0.13
VO_2peak_ *T*2	1.05 (±0.36)	0.45 (±0.24)	0.002^**∗**^
METS *T*1	6.38 (±8.40)	3.74 (±1.10)	0.45
METS *T*2	4.93 (±1.97)	2.64 (±1.28)	0.023^**∗**^
RCP *T*1	0.88 (±0.15)	0.83 (±0.08)	0.45
RCP *T*2	0.86 (±0.13)	1.22 (±0.90)	0.42
VCO2 *T*1	1.01 (±0.41)	0.69 (±0.27)	0.086
VCO2 *T*2	0.95 (±0.40)	0.42 (±0.19)	0.011^**∗**^
VE *T*1	32.8 (±11.4)	24.06 (±7.3)	0.088
VE *T*2	31.4 (±11.27)	17.8 (±5.21)	0.019^**∗**^
HRmax1	87.8 (±27.08)	95.2 (±17.1)	0.5
HRmax2	96.06 (±20.8)	102.0 (±14.8)	0.49

*T*1: start of study, *T*2: end of study. VO_2_ predicted *T*1, VO_2_ predicted at start of study; % VO_2_ predicted *T*1, % VO_2_ predicted at start of study; VO_2_ peak (oxygen uptake at peak effort peak), VCO_2_ (carbon dioxide output), METS (metabolic equivalent), RCP (respiratory compensation point), VE (minute ventilation) expressed in L/min, and HR max (heart rate maximum) expressed in beats/minute. ^*∗*^Significant results (*p ≤ *0.05) are represented in bold.

**Table 4:** Cardiopulmonary exercise testing measurements at the anaerobic threshold (AT). *T*-test.

CPET variables (L/min)	Group 1 (*n* = 24)	Group 2 (*n* = 6)	*p* value *t*-test (G1-G2)
	(mean ± sd)	(mean ± sd)	
VO_2AT_*T*1	0.80 (0.25)	0.83 (±0.28)	0.8
VO_2AT_ *T*2	0.79 (±0.29)	0.40 (±0.15)	0.02^**∗**^
METS *T*1	3.62 (±1.7)	3.77 (±0.95)	0.8
METS *T*2	3.54 (±1.38)	2.15 (±0.97)	0.07
VCO_2_*T*1	0.70 (±0.27)	0.71 (±0.29)	0.9
VCO_2_*T*2	0.68 (±0.35)	0.30 (±0.14)	0.03^**∗**^
VE *T*1	22.6 (±6.40)	23.3 (±8.17)	0.8
VE *T*2	25.2 (±12.2)	14.62 (±2.85)	0.10

*T*1: admission, *T*2: end of study. VO2 AT (oxygen uptake peak at anaerobic threshold), VCO2 (carbon dioxide output), METs (metabolic equivalent), and VE (minute ventilation) are expressed in L/min. ^*∗*^*Significant results *(*p ≤ *0.05)* are represented in bold.*

**Table 5:** Correlations between ALSFRS-R total score and CPET variables on the peak of effort at *T*2.

CPET variables (L/min)	ALSFRS- R total score in *T*2	
	Pearson correlation	Sig. (2-tailed)
METS	**0.491**	0.017^*∗*^
RCP	0.256	0.239
VCO_2_	**0.580**	0.004^*∗*^
VE	**0.585**	0.003^*∗*^
VO_2Peak_	**0.544**	0.007^*∗*^

ALSFRS-R (ALS functional scale revised), METs (metabolic equivalent), VCO2 (carbon dioxide output), RCP (respiratory compensation point), VO2 peak (oxygen uptake peak), and VE (minute ventilation) expressed in L/min. ^*∗*^*Significant results *(*p ≤ *0.05)* are represented in bold.*

## Abbreviations

ALS: Amyotrophic Lateral Sclerosis
ALSFRS-R: Revised ALS Functional Rating Scale
AT: Anaerobic Threshold
BWSS: Body Weight Supporting System
CPET: Cardiopulmonary exercise testing
FVC: Forced vital capacity
NIV: Noninvasive ventilation
NPO: Nocturnal pulsed oximetry
NS: Nonsignificant
RFT: Respiratory Function Tests
ROM: Range of motion
RCP: Respiratory Compensation Point
VO2: Oxygen uptake
VO2pk: Oxygen uptake at peak effort
VT1: First ventilatory threshold
VT2: Second ventilatory threshold.
